# Disinfection By-Products Formation from Chlor(*am*)ination of Algal Organic Matter of *Chlorella sorokiniana*

**DOI:** 10.3390/toxics11080690

**Published:** 2023-08-10

**Authors:** Luan de Souza Leite, Danilo Vitorino dos Santos, Cristina Filomena Pereira Rosa Paschoalato, Tom Bond, Luiz Antonio Daniel

**Affiliations:** 1 Department of Hydraulics and Sanitation, São Carlos School of Engineering, University of São Paulo, Av. Trabalhador São-Carlense, 400, São Carlos 13566-59, São Paulo, Brazil; 2 School of Sustainability and Civil Engineering, University of Surrey, Guildford GU2 7XH, UK; 3 Laboratory of Chemical Residues, University of São Paulo, Ribeirão Preto 05508-220, São Paulo, Brazil; 4 Department of Environmental Technology, University of Ribeirão Preto (UNAERP), Ribeirão Preto 14096-900, Brazil

**Keywords:** bromide, chlorination, chloramination, trihalomethanes

## Abstract

Eutrophication in water reservoirs releases algal organic matter (AOM), which is an important precursor of disinfection by-products (DBPs) formed during water treatment. *Chlorella sorokiniana* is a microalgae which flourishes under conditions of high light intensity and temperature, thus its prevalence in algal blooms is expected to increase with climate change. However, *Chlorella sorokiniana* AOM has not been previously investigated as a DBP precursor. In this context, this study evaluated the effect of AOM concentration, humic acid (HA), and pH on DBP formation from chlor(*am*)ination of AOM *Chlorella sorokiniana*. DBP yields determined by linear regression for trichloromethane (TCM) and chloral hydrate (CH) were 57.9 and 46.0 µg·mg DOC^−1^ in chlorination, while the TCM, CH, dichloroacetonitrile (DCAN), 1,1,1-trichloropropanone (1,1,1-TCP), and chloropicrin (CPN) concentrations were 33.6, 29.8, 16.7, 2.1, and 1.2 µg·mg DOC^−1^ in chloramination. Chloramination reduced the formation of TCM and CH but increased CPN, DCAN, and 1,1,1-TCP yields. AOM *Chlorella sorokiniana* showed a higher DBP formation than 9 of 11 algae species previously investigated in the literature. At basic pH, the concentration of TCM increased while the concentration of other DBP classes decreased. Bromide was effectively incorporated into the AOM structure and high values of bromine incorporation factor were found for THM (1.81–1.89) and HAN (1.32) at 1.5 mg Br·L^−1^. Empirical models predicted successfully the formation of THM and HAN (*R*^2^ > 0.86). The bromide concentration had more impact in the model on the DBP formation than AOM and HA. These results provide the first insights into the DBP formation from AOM chlor(*am*)ination of *Chlorella sorokiniana*.

## 1. Introduction

Human activities, such as agriculture, industrialization, and urbanization, have contributed to the global occurrence of eutrophication. The phenomenon has been becoming increasingly frequent and severe over time as a result of climate change [1,2]. Algal blooms, including those of cyanobacteria, diatoms, and green microalgae, can greatly degrade water quality by raising pH levels, increasing turbidity, and reducing dissolved oxygen concentrations [3,4]. Algal organic matter (AOM) is released as algae proliferate through both metabolic processes and cell lysis, and it is comprised of a variety of compounds including carbohydrates, lipids, proteins, nitrogen-containing compounds (amino acids, peptides, nucleic acids), and various organic acids [5,6,7]. AOM creates significant difficulties in the operation of drinking water treatment [8]. Conventional clarification processes (i.e., coagulation-flotation flotation or sedimentation, then rapid sand filtration) applied in drinking water facilities are not effective at removing the AOM, with optimised removal efficiencies from 25 to 71% reported [7,9]. Therefore, AOM can persist in downstream treatment processes, including chemical disinfection by chlor(*am*)ination, leading to the formation of undesirable DBPs.

AOM is a well-known precursor of DBPs, such as chloral hydrate (CH), haloacetonitriles (HANs), and trihalomethanes (THMs) [10,11,12]. Research into the formation of DBPs from the AOM chlor(*am*)ination has been prioritized since AOM is recognized as one of the major precursors of DBP formation. Together algae and AOM can contribute from 20 to 50% of the DBP formed during algal blooms [13]. Carbonaceous DBPs (C-DBPs), such as THM and haloacetic acids (HAA) [14,15,16], can represent more than 50% by weight of total DBPs from AOM [14,17]. THMs and HAAs are widely regulated by public health authorities regulated, for example, at 100 and 60 µg·L^−1^ in Europe [18]. Investigations about the formation of nitrogenous DBPs (N-DBPs) have increased in recent years, as these DBPs can have greater genotoxicity and cytotoxicity than C-DBPs [19]. They are not regulated but some limits are suggested in the WHO drinking water guidelines, for example, dichloroacetonitrile (DCAN) and dibromoacetonitrile (DBAN) at 20 and 70 µg·L^−1^, respectively [20]. 

Research has been conducted to examine the DBP formation from AOM chlor(*am*)ination of different algae species, such as *Anabaena flos-aquae*, *Aphanizomenon flos-aquae*, *Asterionella Formosa*, *Chlorella vulgaris*, *Chlorella* sp., *Dolichospermum circinale*; *Microcystis aeruginosa*, *Chaetoceros mulleri*, *Melosira* sp., *Oscillatoria prolifera*, *Scenedesmus quadricauda*, and *Scenedesmus subspicatus* [17,21,22,23,24,25,26,27,28]. In addition, samples collected from the environment with a variety of algae species were also analyzed [29,30]. For example, DBP formation from chlor(*am*)ination of drinking water source (Taihu Lake, China) (main species *Microcystus* sp. and *Anabaena* sp.) [29] and Red Sea (main specie *Synecococcus* sp.) [30] containing AOM were reported. The cyanobacteria *Microcystis aeruginosa* is the most analyzed specie due to its abundance in eutrophication events [25]. Despite the extensive research, certain algal species, including members of *Chlorella* genus other than *Chlorella vulgaris*, have been overlooked even though they can be the prevalent species in algal blooms [31,32]. The freshwater microalgae species *Chlorella sorokiniana* is found worldwide [33,34,35] and, in contrast to other *Chlorella* species, can flourish under conditions of high light intensity (2100 µmol·m^−2^·s^−1^) [36] and temperature (38–42 °C) [37]. These characteristics make this species of particular interest, given its prevalence in algal blooms may be expected to increase with climate change. Given this background, this study aims to evaluate the effect of (1) AOM concentration; (2) HA concentration; (3) pH; (4) bromide concentration; and (5) the effect of multiple compounds on the DBP formation from chlor(*am*)ination of AOM of *Chlorella sorokiniana*. To our best knowledge, it is the first the *Chlorella sorokiniana* AOM has been investigated regarding DBP formation.

## 2. Materials and Methods

### 2.1. Materials and Methods

*Chlorella sorokiniana* 211-8k was obtained from the Culture Collection of Algae and Protozoa, Argyll, Scotland. *Chlorella sorokiniana* was cultivated according to Leite et al. (2021) [38]. The cells were collected at the stationary growth phase reached after seven days of cultivation. Then, the suspension was centrifuged (1500× *g*, 10 min), washed twice with ultrapure water, and frozen at −20 °C.

The AOM from *Chlorella sorokiniana* cells was extracted using the protocol suggested by Leite et al. (2019) [39]. In brief, the cells were resuspended in ultrapure water and ultrasonicated twice in ice. Subsequently, the suspension was centrifuged (1500× *g*, 10 min) and the supernatant was filtered by a 0.45 µm membrane. Then, the extracted AOM was kept at −20 °C until use. The dissolved organic carbon (DOC) content of the AOM was quantified using a TOC-L analyser (Shimadzu, Kyoto, Japan).

### 2.2. Chlor(am)ination Tests and DBP Quantification

The chlor(*am*)ination protocol was adapted from method 5710 [40]. Experiments were undertaken in amber glass with 20 mL samples, buffered at pH 8 (20 mg CaCO_3_·L^−1^ of alkalinity), a mass ratio of 5:1 mg Cl_2_∙mg DOC^−1^, the contact time in the dark of 7 days, and temperature of 20 °C. The alkalinity and pH values were selected based on the water quality found in eutrophicated reservoirs in São Paulo State, Brazil [38]. The formation of chloramine was induced by the addition of ammonium sulfate (Sigma-Aldrich, Saint Louis, MO, USA) as a nitrogen source in the solution. The chloramine was formed at the optimum mass ratio of 5:1 Cl_2_:NH_3_-N [41] by mixing the free chlorine added and ammonia in the solution. At the end of the contact time, solutions were quenched with ascorbic acid (Qhemis, Jundiaí, São Paulo, Brazil) at a mass ratio of 6:1 as optimized in preliminary tests. Then, 5 mL of samples were extracted immediately using 5 mL of MTBE (Sigma-Aldrich, Saint Louis, MO, USA) following EPA 551.1 method [42].

The concentration of free and total chlorine was determined by DPD (N,N-diethyl-p-phenylenediamine) colourimetric method using powder pillows (Hach, Ames, IA, USA). The concentration of residual disinfectant at the end of tests was always above 2.0 mg Cl_2_∙L^−1^ ensuring it did not limit the DBP formation.

The presence of target DBPs in the chlor(*am*)ination experiments was assessed in this study, including THMs (trichloromethane (TCM), bromodichloromethane (BDCM), dibromochloromethane (DBCM), and tribromomethane (TBM)), HANs (bromochloroacetonitrile (BCAN), dibromochloroacetonitrile (DBCAN), and DCAN, trichloroacetonitrile (TCAN)), HKs (1,1-dichloropropanone (1,1-DCP) and 1,1,1-trichloropropanone (1,1,1-TCP)), chloropicrin (CPN), and CH. Their concentrations were quantified by a gas chromatograph- electron capture detector (GC-2010, Shimadzu, Kyoto, Japan) with polysiloxane column (DB-1, Agilent J&W, Santa Clara, CA, USA) following USEPA 551 method [42]. Each experiment was performed in duplicate and blank tests were undertaken using ultrapure water.

A mixed standard of EPA 551B Halogenated Volatiles Mix (Supelco, Sigma-Aldrich, Saint Louis, MO, USA) and EPA 501/601 Trihalomethanes Calibration Mix (Supelco, Sigma-Aldrich, Saint Louis, MO, USA), and CH (Dinâmica, Indaiatuba, São Paulo, Brazil) were used to construct the calibration curves. The calculated limits of detection (LD) were ≤ 0.7 µg·L^−1^ for THMs, ≤ 1.5 µg·L^−1^ for HANs, ≤ 1.8 µg·L^−1^ for HKs, ≤ 3.2 µg·L^−1^ for CH, and ≤ 3.3 µg·L^−1^ for CPN. The DBP yields (µg·mg DOC^−1^) were normalized to DOC dividing the DBP concentration (µg·L^−1^) by the AOM concentration (mg DOC·L^−1^).

### 2.3. Experimental Design

In total, the impact of 25 different experimental conditions of a single factor on DBP formation was evaluated (Table 1), encompassing a range of AOM, humic acid (HA) and bromide (Br^−^) concentrations, and pH values. The selected values represent a typical range of pH [43,44], organic carbon concentration [45,46], and bromide concentration [20,47,48] found in surface waters. 

The pH of the water test was modified using 1 N NaOH or 1 N HCl (Qhemis, Jundiaí, São Paulo, Brazil). HA solution was prepared by diluting 0.5 g of HA (Sigma-Aldrich, Saint Louis, MO, USA) in 1 L ultrapure water, mixing for 2 h and filtered using a 0.45 μm membrane. HA concentration was also expressed as DOC value. Required bromide concentration was added from a standard solution of KBr (1000 mg Br·L^−1^).

To simulate the complexity of real water matrices, the effect of the multiple factors on DBP formation previously tested individually (HA + Br + AOM) was also assessed in four levels (i.e., C0, C1, C2, C3, and C4) (Table 1).

### 2.4. Bromine Incorporation Factor (BIF)

The BIF quantifies the amount of bromine incorporated into a DBP class as a proportion of the total formation of chlorinated and brominated DBPs. It is a useful parameter to compare the degree of bromination of the DBP classes in chlor(*am*)ination, since brominated DBPs are more genotoxic and cytotoxic than chlorinated DBPs [49]. The BIFs for THM and HAN classes were calculated using Equations (1) and (2), where concentrations are on a molar basis. BIFs values vary between 0 (no formation of brominated species) to 3 and 2 (only formation of tribrominated (THMs) or dibrominated (HANs) species) for THMs and HANs, respectively, depending on the degree of bromine incorporation.

(1) $$BIF\;(THMs)=\frac{\left[{CHBrCl}_{2}\right]+2\cdot \left[{CHBr}_{2}Cl\right]+3\cdot \left[{CHBr}_{3}\right]}{\left[{CHCl}_{3}\right]+\left[{CHBrCl}_{2}\right]+\left[{CHBr}_{2}Cl\right]+\left[{CHBr}_{3}\right]}$$

(2) $$BIF\;(HANs)=\frac{\left[C_{2}HBrClN\right]+2\cdot \left[C_{2}H{Br}_{2}N\right]}{\left[C_{2}H{Cl}_{2}N\right]+\left[C_{2}{Cl}_{3}N\right]+\left[C_{2}HBrClN\right]+\left[C_{2}H{Br}_{2}N\right]}$$

### 2.5. Statistical Analysis

Correlations between DBPs yield and relevant water quality parameters were assessed by linear regression. The DBP concentrations per mg of AOM/HA were reported based on the slope of the equations generated by a linear equation of each data set (*n* = 7–8). Experimental data of THM and HAN were modelled using empirical models (linear, exponential, and logarithmic equations), as summarised by Chowdhury et al. (2009) [50] using both linear and non-linear regression. The fitness of the model to the data was analyzed by the coefficient of determination (*R*^2^). Modelling was undertaken using Microsoft Excel Solver.

## 3. Results and Discussion

### 3.1. Impact of AOM Concentration on DBP Formation

**Figure 1:**
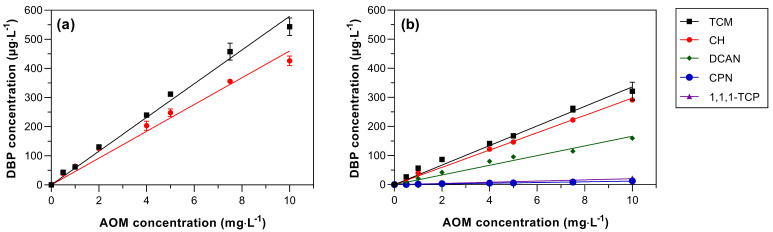
The effect of AOM concentration in DBP formation from (**a**) chlorination and (**b**) chloramination. Experimental conditions: Cl_2_:DOC = 5:1, pH = 8.0, temperature = 20 °C, and reaction time = 7 d. Error bars are the standard deviations of duplicate samples.

TCM and CH were the DBPs detected following chlorination in the absence of bromide, while TCM, CH, DCAN, CPN, and 1,1,1-TCP were quantified following chloramination (Figure 1). Thus, TCM, DCAN and 1,1,1-TCP were the only types of THM, HAN and HK detected, respectively, following the application of both chlorine and chloramine. Yields of all DBPs increased with increasing AOM concentration using both disinfectants (Figure 1). Relationships between AOM concentration and DBP formation showed high linearity for all DBPs (Figure 1), as illustrated by *R*^2^ > 0.98 for all DBPs. Based on the regression lines, yields of TCM and CH were 57.9 and 46.0 µg·mg DOC^−1^ following chlorination, while the TCM, CH, DCAN, 1,1,1-TCP, and CPN concentrations were respectively 33.6, 29.8, 16.7, 2.1, and 1.2 µg·mg DOC^−1^ following chloramination. 

These values are consistent with previous studies of AOM chlor(*am*)ination of different algae species and testing conditions (Table 2), in which the concentration range usually follows the order of THM (0–176.8 µg·mg DOC^−1^) > CH (0.2–32.5 µg·mg DOC^−1^) > HAN (0–62.5 µg·mg DOC^−1^) > HK (0.0–77.5 µg·mg DOC^−1^) > CPN (0–27.5 µg·mg DOC^−1^) [17,21,22,23,24,25,26,27,28]. This is not a direct comparison, because experimental conditions vary between the different studies. For example, most literature studies were performed at pH 7 while the present study was undertaken at pH 8. However, AOM *Chlorella sorokiniana* showed a higher DBP formation than 9 of 11 algae species previously investigated in the literature (Table 2). The CH concentration (46 µg·mg DOC^−1^) following chlorination experiments in the current work is higher than literature values (Table 2). This is likely because CH is an intermediate by-product, which can decompose to TCM and trichloroacetic acid at varying speeds, depending on reaction conditions [51]. Therefore, CH concentrations are very sensitive to the experimental conditions used. 

Chloramination reduced the formation of some DBP species (42.0% of TCM and 35.2% of CH) but promoted the formation of N-DBPs (HAN and CPN) and 1,1,1-TCP. The lower concentration of these species in chloramination than in chlorination is consistent with previous studies, though the magnitude of the reduction varies between studies and experimental conditions [25,26,30]. 

The absence of N-DBPs during chlorination indicates that the inorganic nitrogen present in chloramine was responsible for the formation of nitrogenous species. Previous studies also reported chloramine acts as a precursor for some N-DBPs (e.g., cyanogen chloride, N-nitrosodimethylamine, and trichloronitromethane) during AOM chloramination, even if the precise formation pathway are sometimes unclear [17,26]. 

### 3.2. Effect of pH on DBP Formation

**Figure 2:**
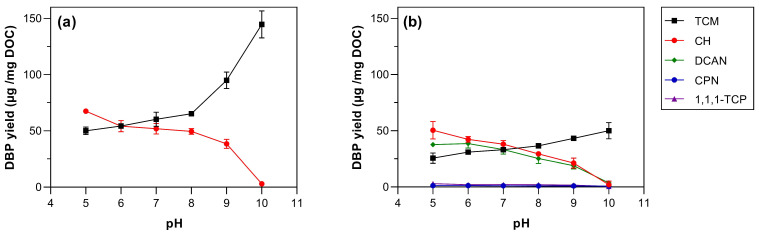
The pH effect (pH 5 to 10) in DBP formation from (**a**) chlorination and (**b**) chloramination. Experimental conditions: Cl_2_:DOC = 5:1, AOM = 5·mg DOC L^−1^, temperature = 20 °C, and reaction time = 7 d. Error bars are the standard deviations of duplicate samples.

TCM concentration increased at high pH, reaching the highest concentration of 50.0 and 144.8 µg·mg DOC^−1^ at pH 10 for chloramination and chlorination, respectively (Figure 2). This reflects the importance of base-catalysed reaction steps in THM formation, such as the hydrolysis of haloacetic acids, haloketones (e.g., 1,1,1-TCP), and haloaldehydes to generate THMs [52]. Conversely, the concentration of other DBPs decreased with increasing pH (Figure 2). In general, N-DBPs and CH had maximum yields for both disinfectants at pH 5–6. For example, the highest CH yield of 50.40 and 67.47 µg·mg DOC^−1^ was found at pH 5 for chloramination and chlorination, respectively. The N-DBPs reached their maximum yield at different pH, for example, 38.6 µg·mg DOC^−1^ DCAN at pH 6, 1.2 µg·mg DOC^−1^ CPN at pH 5, and 3.0 µg·mg DOC^−1^ 1,1,1-TCP at pH 5 (all data following chlorination/chloramination). Similar trends in the DBP formation were also reported by a previous study of AOM chlorination of *Microcystis aeruginosa*. Fang et al. (2010a) [15] observed that the pH variation from 6.0 to 9.0 increased the THM yield and decreased CH, HK, and HAN yields. 

The pH affects the chlor(*am*)ination reactivity with the AOM and the stability of the DBPs formed in the solution. Hydrolysis decomposition of unstable DBPs (e.g., 1,1,1-TCP, CH, and DCAN) has higher rates at basic pH [53]. TCM can also be formed by the hydrolysis of 1,1,1-TCP, CH, and trichloroacetic acid [51,54]. Therefore, TCM concentration increased and the unstable DBPs species decreased at basic pH values as observed in the results (Figure 2).

### 3.3. Effect of Humic Acid (HA) Concentration on DBP Formation

**Figure 3:**
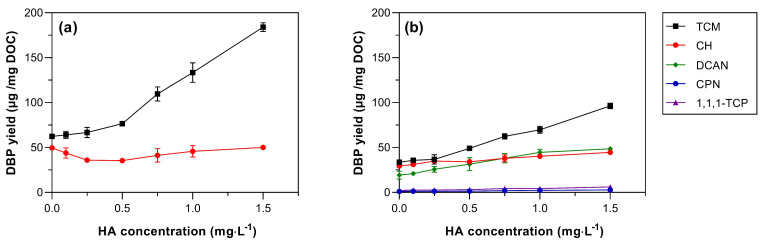
The impact of humic acid (HA) concentration (from 0 to 1.5 mg DOC·L^−1^) on DBP formation in the AOM solution from (**a**) chlorination and (**b**) chloramination. Experimental conditions: Cl_2_:DOC = 5:1, AOM = 5·mg DOC L^−1^, pH = 8.0, temperature = 20 °C, and reaction time = 7 d. Error bars are the standard deviations of duplicate samples.

The NOM is the major precursor of DBP formation and is primarily comprised of HA and fulvic acid. The effect of HA, a widely used surrogate for NOM in studies on AOM [55,56], on DBP formation was evaluated (Figure 3). Following chlorination, HA addition increased TCM yields (359.19 µg·mg DOC^−1^) while CH remained essentially constant. Following chloramination, all the DBP species increased with the increasing HA concentration. For instance, 194.2 µg·mg DOC^−1^ of TCM, 53.4 µg·mg DOC^−1^ of CH, 111.0 µg·mg DOC^−1^ of HAN, 12.9 µg·mg DOC^−1^ of 1,1,1-TCP, and 5.0 µg·mg DOC^−1^ of CPN were the concentrations per mg HA generated in the presence of 0.1–1.5 mg DOC L^−1^ of HA (Figure 3). These values are higher than some previous data relating to the DBP formation from natural organic matter. For example, the range of THM yield has been reported to range from 20 to 281 µg·mg TOC^−1^ for natural water sources [21,57,58]. This difference may happen due to the different testing conditions such as chlorine dose (1:1 to 1:5 Cl_2_:DOC/TOC), contact time (3 to 7 d), pH (6–7.5), and organic matter present (AOM, fulvic acid, and HA), and a potential synergistic effect when both HA and AOM are present in the solution. In general, the chloramination reduced the TCM and CH yield by 35.9–47.8% and 2.8–29.3%, respectively, compared to the chlorination. These results indicate that although AOM may not be the biggest contributor to the formation of DBP compared to NOM, it still has a significant contribution to the DBP yield.

### 3.4. Effect of Bromide Concentration on DBP Formation

**Figure 4:**
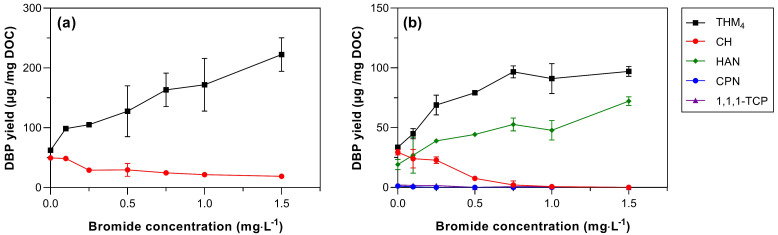
The effect of Br concentration (from 0 to 1.5 mg Br·L^−1^) on DBP formation from (**a**) chlorination and (**b**) chloramination. Experimental conditions: Cl_2_:DOC = 5:1, AOM = 5·mg DOC L^−1^, pH = 8.0, temperature = 20 °C, and reaction time = 7 d. Error bars are the standard deviations of duplicate samples. Quantified brominated DBPs were three THMs (bromodichloromethane, dibromochloromethane, and tribromomethane) and two HANs (dibromochloroacetonitrile and bromochloroacetonitrile).

Bromide addition increased the concentration of brominated types of HANs and THMs, as well as the overall yields of these groups (Figure 4). Yields of chlorinated DBP decreased while their brominated analogues increase. For example, the TCM concentration following chlorination decayed from 62 µg·mg DOC^−1^ at 0·mg Br L^−1^ to 0 µg·mg DOC^−1^ at 1.5·mg Br L^−1^. THM and HAN formation following chloramination reached concentrations of 97 and 72 µg·mg DOC^−1^ for THMs and HANs at 1.5 mg Br L^−1^, respectively. Chloramination in the presence of bromide reduced the THMs and CH formation by 34.4–56.3% and 21.5–100% compared to chlorination. These findings align with the results of other studies, which have also demonstrated that using chloramination can decrease the formation of DBPs even in the presence of bromide [27,59].

**Figure 5:**
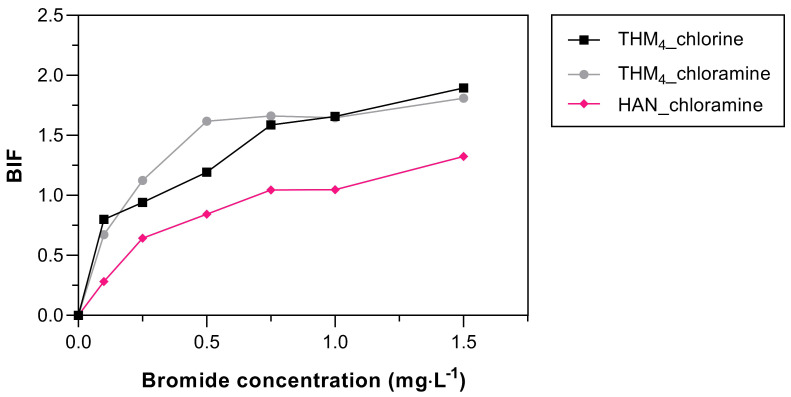
The effect of BIF values when the Br concentration varied from 0 to 1.5 mg·L^−1^.

BIF results are shown in Figure 5. Chloramination and chlorination had similar results reaching plateau values at high bromide concentrations. The maximum BIF results for THM were 1.89 and 1.81 at 1.5 mg·L^−1^ for chlorination and chloramination, respectively, while the BIF of HAN was 1.32 at the same bromide dose. These values are indicative of high bromine incorporation (Figure 3). This supports the observation that bromine is more efficiently incorporated into low UV-absorbing (i.e., low SUVA) compounds such as AOM (SUVA of 0.6 L·m^−1^·mg^−1^) as observed in other studies [60].

Similar results were observed in THMs formed from AOM chlorination of *Chlorella vulgaris* (BIF = 1.4) and *Microcystis aeruginosa* (BIF = 1.4) with 1.0 mg Br·L^−1^ [61]. While Chen et al. (2017) [27] found BIF values for THM formed from AOM *Microcystis aeruginosa* of 0.51–0.68 for chlorination and 0.38–0.87 for chloramination with 0.5 mg Br·L^−1^. The incorporation of bromide in the structure of AOM depends on the algae species and the Br/DOC ratios [59,61], which may explain the differences between our results and the previous studies.

### 3.5. Multiple Parameters

**Figure 6:**
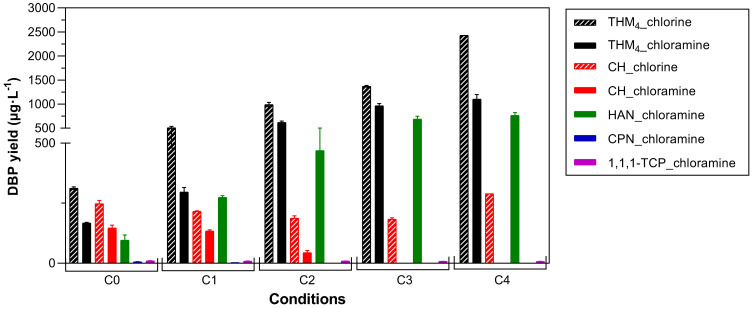
DBP formation under different testing conditions. Experimental conditions: Cl_2_:DOC = 5:1, pH = 8.0, temperature = 20 °C, and reaction time = 7 d. *(C0)* 5 mg DOC·L^−1^ AOM, *(C1)* 5 mg DOC·L^−1^ AOM + 0.1 mg DOC·L^−1^ HA + 0.1 mg·L^−1^ Br, (*C2*) 5 mg DOC·L^−1^ AOM + 0.5 mg DOC·L^−1^ HA + 0.5 mg·L^−1^ Br, (*C3*) 5 mg DOC·L^−1^ AOM + 1.0 mg DOC·L^−1^ HA + 1.0 mg·L^−1^ Br, and (*C4*) 5 mg DOC·L^−1^ AOM + 1.5 mg DOC·L^−1^ HA + 1.5 mg·L^−1^ Br. Error bars are the standard deviations of duplicate samples.

The effect of the multiple parameters on DBP formation previously tested individually (HA + Br + AOM) was also assessed simultaneously to simulate the complexity of real water matrices (Figure 6). The results support the previous data indicating that the higher presence of additional precursors (HA, Br) increased significantly the DBP yield. The scenario with only AOM present (C0) formed 312.3 and 248.2 µg·L^−1^ for THMs and CH after chlorination, respectively, and 167.8, 146.8, 95.9, 10.3, and 5.8 µg·L^−1^ for THMs, CH, HANs, 1,1,1-TCP, and CPN after chloramination. Meanwhile, the worst case scenario (C4, AOM + HA + Br) formed 2430.7 and 289.6 µg·L^−1^ for THMs and CH following chlorination, respectively, and 1106.2, 765.9, and 6.9 µg·L^−1^ for THMs, HANs, and 1,1,1-TCP following chloramination. Considering all conditions, chloramination reduced THM and CH formation by 29.5–54.5% and 38.0–100% compared with chlorination.

These conditions show the potential for DBPs to exceed regulatory limits (e.g., 100 µg·L^−1^ for THM_4_ in the EU [18] and WHO guidelines (300 µg·L^−1^ for TCM, 100 µg·L^−1^ for TBM and DBCM, 100 µg·L^−1^ for BDCM, 20 µg·L^−1^ for DCAN, and 70 µg·L^−1^ for DBAN) [20]. While it should be noted that this study did not record DBP formation under conditions identical to those found in full-scale water treatment (e.g., concentrations of *Chlorella sorokiniana* were higher) this does nonetheless highlight the potential for this species of algae to generate significant concentrations of DBPs. In turn, the study indicates that *Chlorella sorokiniana* blooms can threaten drinking water quality and this species’ presence should be therefore monitored and mitigated. It emphasises the importance of removing AOM before the addition of chemical disinfectants during drinking water treatment.

### 3.6. Modeling DBP Yields

Modeling of DBP yields is a helpful tool to quantify and understand the influence of key water quality parameters on DBP formation. Various empirical models have been reported in the literature to predict the impacts of water quality parameters on DBP yields as summarised by Chowdhury et al. (2009) [50]. For example, different water quality parameters (pH, bromide, DOC, contact time, temperature, etc) and types of equations (linear, quadratic, polynomial, and exponential equations) had been used to predict the DBP formation. Typical types of equations were tested to the data obtained in this study at pH 8, incorporating the water quality parameters evaluated: AOM, Br, and HA concentration (mg·L^−1^). The most appropriate model to predict the DBP yield (µg·L^−1^) had the following general form:

(3) $$DBP=a\left[AOM\right]+b\left[HA\right]+c[Br]$$

where *a*, *b*, and *c* are the model constants.

The data were modelled by linear regression. THM model (*R*^2^ = 0.95) had a greater fit (Equation (4)) to the chlorination data, meanwhile, THM (*R*^2^ = 0.91, Equation (5)) and HAN (*R*^2^ = 0.86, Equation (6)) had a greater fit to the chloramination data.

(4) $$THM=54.86\left[AOM\right]+503.28\left[HA\right]+714.59[Br]$$

(5) $$THM=33.78\left[AOM\right]+263.05\left[HA\right]+350.45[Br]$$

(6) $$HAN=18.18\left[AOM\right]+183.07\left[HA\right]+255.43[Br]$$

The effective THM and HAN models highlights the utility of simple empirical models for predicting DBP yields when AOM is present in the solution. The three constants are positive, indicating THM and HAN yields increased with each parameter. In terms of relative weighting, changes in bromide concentration had more impact on the DBP formation than either HA or AOM. The modeling results emphasize the greater reactivity of bromine versus chlorine in generating THMs and HANs, as evident in previous results (Section 3.4). This reflects the higher efficiency of bromine in participating in halogen substitution reactions than chlorine [47]. The constants of the THM model for chlorination data are higher than the ones from the model of chloramination data, re-emphasizing the higher DBP production following chlorination observed. 

No previous study reported models with the same equation form and variables for AOM chlor(*am*)ination to compare with ours. Ersan et al. (2021) [62] developed a model using literature data to predict HAN formation from AOM chlorination, the logarithmic equation included other variables such as absorbance at 254 nm, pH, chlorine dose, and time. Hua et al. (2018) [63] modelled HAA and THM formation from AOM chlorination of *Chlorella* sp. using AOM properties (i.e., peak sizes from molecular characterization) and obtained an exponential equation. 

### 3.7. Discussion: Implications for Water Treatment

This study has shown *Chlorella sorokiniana* is a more potent DBP precursor than 80% of algae previously investigated in literature. Considering its prevalence in algal blooms is expected to increase due to climate change, it is important to develop control strategies for reactive algae such as this one. The modeling results suggest bromide removal would perhaps be the most effective approach, but currently, there are no efficient/economic treatment options for bromide removal [64]. Conventional treatment processes (coagulation-flotation flotation or sedimentation) resulted in AOM removal typically <71% under optimized bench-scale conditions [7,9]. Thus, additional processes are required to improve AOM removal downstream of clarification methods and consequently the DBP formation in chlor(*am*)ination. Oxidation processes are inherently a high-risk approach as they have the potential to increase downstream DBP formation. In particular, ozonation (22–781%) [14,26,65] and UV irradiation (5–95%) [56,66] of AOM have been reported to enhance the formation of DBPs produced by downstream chlor(*am*)ination. Therefore, physical removal processes, for example, ultrafiltration and/or activated carbon adsorption, are expected to be the most pragmatic and effective methods.

## 4. Conclusions

This study investigated the effect of AOM concentration, HA, and pH on the DBP formation from chlor(*am*)ination of AOM *Chlorella sorokiniana*. DBP yields determined by linear regression for TCM and CH were 57.9 and 46.0 µg·mg DOC^−1^ following chlorination, while mean concentrations of TCM, CH, DCAN, 1,1,1-TCP, and CPN concentrations were 33.6, 29.8, 16.7, 2.1, and 1.2 µg·mg DOC^−1^, respectively during chloramination. Chloramination reduced the formation of TCM and CH but increased CPN, DCAN, and 1,1,1-TCP yields. At basic pH, the concentration of TCM increased while the concentration of other DBP classes decreased. Bromide was effectively incorporated into the AOM structure and high BIF values were found for THM (1.81–1.89) and HAN (1.32) at a bromide dose of 1.5 mg·L^−1^. Empirical models were generated using the data obtained and effectively predicted the formation of THMs and HANs (*R*^2^ > 0.86). Changes in bromide concentration had more impact on the DBP formation than AOM and HA in the model. These results provide the first insights into the DBP formation from AOM chlor(*am*)ination of *Chlorella sorokiniana*, which was found to be more reactive as a DBP precursor than 80% of algae previously investigated in literature. Nonetheless, removing AOM and natural organic matter before disinfection is anticipated to be an effective control strategy for this species of algae. 

## Figures and Tables

**Table 1:** Summary of experimental conditions used in the study.

AOM Concentration (mg DOC·L^−1^)	pH	HA Concentration (mg DOC·L^−1^)	Bromide Concentration (mg Br·L^−1^)
0.5, 1.0, 2.0, 4.0, 5.0, 7.5, and 10	5, 6, 7, 8, 9, and 10	0.10, 0.25, 0.50, 0.75, 1.00, and 1.50	0.10, 0.25, 0.50, 0.75, 1.00, and 1.50
**Conditions**	**Composition**		
C0	5 mg DOC·L^−1^ AOM		
C1	5 mg DOC·L^−1^ AOM + 0.1 mg DOC·L^−1^ HA + 0.1 mg Br·L^−1^		
C2	5 mg DOC·L^−1^ AOM + 0.5 mg DOC·L^−1^ HA + 0.5 mg Br·L^−1^		
C3	5 mg DOC·L^−1^ AOM + 1.0 mg DOC·L^−1^ HA + 1.0 mg Br·L^−1^		
C4	5 mg DOC·L^−1^ AOM + 1.5 mg DOC·L^−1^ HA + 1.5 mg Br·L^−1^		

**Table 2:** Disinfection by-product (DBP) values reported for AOM chlor(*am*)ination of different microalgae species.

Algal Species	Disinfection Parameters (Cl_2_:DOC/TOC(w·w^−1^); pH, Temperature, Contact Time)	DBPs (µg·mg DOC^−1^)					References
		THM	CH	HK	HAN	CPN	
*Anabaena flos-aquae*	Chlorination (5:1, 7.0, 20 °C, 7 d)	26–26.6			0.39	0.16	[21,24]
*Aphanizomenon flos-aquae*	Chlorination (5:1, 7.0, 20 °C, 7 d)	56.6			0.12	0.11	[21]
*Asterionella formosa*	Chlorination (5:1, 7.0, 20 °C, 7 d)	18.7			0.53	0.24	[21]
*Chaetoceros mulleri*	Chlorination (5:1, 7.0, 20 °C, 7 d)	30					[22]
*Chlorella vulgaris*	Chlorination (10:1, 7.0, 20 °C, 3 d)	23.7			3.5	2.1	[23]
***Chlorella sorokiniana***	**Chlorination** **(5:1, 8.0, 20 °C, 7 d)**	**57.9**	**46.0**				**This study**
	**Chloramination** **(5:1, 8.0, 20 °C, 7 d)**	**33.6**	**29.8**	**2.1**	**16.7**	**1.2**	
*Melosira* sp.	Chlorination (5:1, 7.0, 20 °C, 7 d)	19.5			0.87	0.36	[21]
*Microcystis aeruginosa*	Chlorination (3–10:1, 7.0, 20–25 °C, 3–7 d)	8–176.8	7.0–27.5	0.0–77.5	0.0–62.5	0.0–27.5	[17,21,24,25,26,27,28]
	Chloramination (3–5:1, 7.0, 20–22 °C, 1–3 d)	0.0–9.0	0.2–32.5	0.36–27.5	0.0–57.5	0.1–22.5	[17,25,26,27]
*Oscillatoria prolifera*	Chlorination (5:1, 7.0, 20 °C, 7 d)	30					[22]
*Scenedesmus quadricauda*	Chlorination (5:1, 7.0, 20 °C, 7 d)	48–64					[22]
*Scenedesmus subspicatus*	Chlorination (5:1, 7.0, 20 °C, 7 d)	19.9			1.10	<LD	[21]

## Data Availability

Data are contained within the article.
